# A whole population-based cohort study of the trajectory of the prevalence and the incidence of mental illness, challenging behaviour, and psychotropic medication prescribing in adults with intellectual disabilities in the Czech Republic between 2010 and 2022

**DOI:** 10.1186/s12888-025-07620-7

**Published:** 2025-11-24

**Authors:** Shoumitro Deb, Jiří Jarkovský, Hana Melicharová, David Holub, Bharati Limbu, Petr Třešňák

**Affiliations:** 1 Department of Brain Sciences, Faculty of Medicine, Imperial College London, 2nd Floor Commonwealth Building, Du Cane Road, London, W12 0NN UK; 2 Institute of Health Information and Statistics of the Czech Republic & Institute of Biostatistics and Analyses, Faculty of Medicine, Masaryk University, Netroufalky 5, Brno, 625 00 Czech Republic; 3 Institute for Medical Humanities, 1st Medical Faculty, Charles University, Karlovo náměstí 40, 128 08 Praha 2, Czech Republic; 4 Parent of an autistic child and the Chairman of the Children of the Full Moon, NGO, Bubenska 6, Prague 8, Czech Republic

**Keywords:** Adults with intellectual disabilities, Mental illness, Challenging behaviour, Psychotropic medications, Neurodevelopmental disorders, Autism, ADHD, Prevalence, Incidence, Cohort study, Czech republic

## Abstract

**Background:**

It is essential to understand the complex interaction among mental illness, challenging behaviour and psychotropic medication prescribing among adults with intellectual disabilities to help reduce overmedication of psychotropics.

**Methods:**

We analysed data from the Institute of Health Information and Statistics of the Czech Republic to estimate the prevalence of mental illness, challenging behaviour and psychotropic prescribing between 2010 and 2022 and incidence between 2015 and 2022.

**Results:**

62,636 (54% males) adults with intellectual disabilities contributed 704,503 person-years of data. In 2010, the prevalence of concomitant mental illness (ICD-10, F20-48) was 15·7%, challenging behaviour 29% and any psychotropic prescription 55%, increasing in 2022 to 17·3%, 30·5% and 59%, respectively. The prevalence of most individual mental illness categories either remained the same or marginally increased between 2010 and 2022, except anxiety disorders, the neurodevelopmental disorders like autism and attention-deficit hyperactivity disorder (ADHD), which showed a significant increase. The incidence of new diagnoses of mental illnesses and challenging behaviour decreased between 2015 and 2022, except for the neurodevelopmental disorders, which increased significantly. The incidence of challenging behaviour correlated significantly with psychoses, bipolar disorder, and anxiety disorder. The overall prevalence of most psychotropic prescribing correlated significantly with the prevalence of mental illnesses and challenging behaviour. Among those receiving antipsychotics, only 18% in 2010 and 19% in 2022 had severe mental illness (psychoses and/or bipolar disorder), which are the licensed indications for long-term antipsychotic prescriptions. In 2010, among those with challenging behaviour, 82% received psychotropics, 62% antipsychotics, 20% antidepressants, 17% anxiolytics, 30% mood stabilisers, and 0·4% hypnotics and sedatives. The incidence rates of new prescriptions among participants who displayed challenging behaviour fell for any psychotropics, antipsychotics, antidepressants and anxiolytics. However, the incidence of new prescriptions increased for mood stabilisers, and significantly for hypnotics/sedatives (Incidence Rate Ratio: 0.61; 95% Confidence Interval: 0.45–0.81; *p* < 0.001).

**Conclusions:**

Challenging behaviour was significantly associated with some mental illnesses. Both mental illness and challenging behaviour were significantly associated with psychotropic medication prescribing. The prevalence of psychotropic medication prescribing overall and especially among those displaying challenging behaviour was high and showed a significant increase in antidepressants and hypnotics/sedatives prescriptions over 12 years.

**Clinical trial number:**

Not applicable.

## Background

About 0·5 − 0·7% of the general adult population have intellectual (learning) disabilities [1–3]. Intellectual disabilities are a group of etiologically diverse conditions originating during the developmental period, characterised by significantly below-average intellectual functioning and adaptive behaviour [4]. Like the general population, adults with intellectual disabilities are prone to a whole range of mental illnesses. However, diagnosing mental illness in adults with intellectual disabilities could be difficult because of the communication challenges and sometimes atypical manifestation, for example, through challenging behaviour [5, 6]. It is, therefore, essential to understand the relationship between mental illness and challenging behaviour in this population using a sizeable, centralised health database. Otherwise, there is a danger of inappropriate overuse of psychotropic prescriptions for challenging behaviour. The overmedication of adults with intellectual disabilities is a significant public health concern [7–10] as the randomised controlled trials (RCT)-based evidence for the efficacy of psychotropics for challenging behaviour in the absence of a mental illness among adults with intellectual disabilities is weak [see 11 for a review], but the evidence of harm caused by the medication’s adverse effects is substantial [12–14]. The World Psychiatric Association (WPA) [15] and the United Kingdom (UK) National Institute for Health and Care Excellence (NICE) [16] have developed guidelines to address this issue, along with the National Health Service (NHS) England initiative STopping Over-Medication of People with learning disabilities, autism or both (STOMP) [17]. All these guidelines recommend the use of non-pharmacological interventions such as positive behaviour support (PBS) [18] first before prescribing psychotropic medications for challenging behaviour. However, there has not been a longitudinal study to assess the impact of these guidelines on psychotropic prescribing for mental illness and challenging behaviour.

Therefore, we studied the change in the prevalence and incidence of mental illness, challenging behaviour, and neurodevelopmental disorders like autism and attention deficit hyperactivity disorder (ADHD), and psychotropic medication prescribing using the International Classification of Diseases-10th revision (ICD-10) [4] in adults with intellectual disabilities between 2010 and 2022 in the Czech Republic to assess the impact of NICE and WPA guidelines and the UK STOMP initiative on psychotropic medication prescribing in this population and its relationship with mental illness and challenging behaviour.

## Methods

We collected data from the National Registry of Reimbursed Health Services (NRRHS) in the Czech Republic. NRRHS contains all healthcare data provided under public health insurance, which covers almost 100% of the population in the Czech Republic. NRRHS is part of the National Health Information System (NHIS) regulated by the Institute of Health Information and Statistics of the Czech Republic (“IHIS CR“ or “the Institute“). The Ministry of Health in the Government of Czechoslovakia established the Institute in 1960 to implement the Health Services Act. The Institute follows the principles of the European Statistics Code of Practice, which represents a common summary of European standards designated for statistical organisations and the whole European statistical system to secure a high quality and credibility of European data. In the Czech Republic, all adults are registered with this national registry to receive reimbursement for their health service costs. The health data such as the diagnosis, treatment and diagnostic tests on all adults in the country who have come across any health service such as in-patient, outpatient or community provided by any specialities such as medicine, surgery, psychiatry, including health professionals like doctors including general practitioners and nurses and other allied health professionals like physiotherapists, psychologists, occupational therapists, radiologists, haematologists, etc. The International Classification of Diseases-10th revision (ICD-10) [4] criteria are used by all who input data into the national register. The prevalence data were collected from 2010, the year after the WPA international guideline [15] was published, until 2022, the last year ICD-10 diagnoses were made, as ICD-11 [19] was published in 2022. The incidence rates of the first occurrence of episodes within the last five years were collected from 2015 to 2022, allowing for the recording of only new entries that were not present prior to 2015.

We have identified adults with intellectual disabilities using the ICD-10 codes of F70-79 from any service, either as a primary or a secondary diagnosis. The intellectual disabilities diagnosis was subdivided into F70 mild intellectual disabilities, F71 moderate intellectual disabilities, F72 severe intellectual disabilities, F73 profound intellectual disabilities, F78 other intellectual disabilities, and F79 unspecified intellectual disabilities. The diagnosis of challenging behaviour was made using the ICD-10 codes F70x.1 (significant impairment of behaviour requiring attention or treatment) and F70x.8 (other impairments of behaviour). A psychiatric diagnosis is recorded using the following ICD-10 codes. F20-29: schizophrenia, schizotypal and delusional disorders (psychoses); F30-31: bipolar disorder; F32-39: depressive disorder; F40-48: anxiety disorders. We used F84 for autism spectrum disorder and F90 for ADHD diagnosis. To compare our findings with the previous English general practice register-based study [8], we recorded a diagnosis of a severe mental illness if someone had a diagnosis of F20-29 (psychosis) and/or F30-31 (bipolar disorder) and also a diagnosis of any mental illness if someone has any of the following ICD-10 diagnosis; F20-48 (psychoses, bipolar disorder, depressive disorder and anxiety disorders).

We have utilised the World Health Organization (WHO) Anatomical Therapeutic Chemical (ATC) codes and the Therapeutic Chemical Classification systems [www.whocc.no/atc-do-index, accessed on 25.03.2025] to classify psychotropic medication groups. The following codes were used. N05A: antipsychotics; NO6A: antidepressants; NO5B: anxiolytics (anti-anxiety medications); NO5C: hypnotics/sedatives; NO3: Anti-epileptic medication; NO5AN: lithium. Anyone receiving NO5AN, NO3AX, NO3AF or NO3AG is recorded as receiving a mood stabiliser. Anyone receiving any of these medications was recorded as receiving any psychotropic.

As the source data did not provide an indication for each psychotropic medication prescribed, we could only calculate the associations between psychotropic prescription and mental illness, and challenging behaviour. However, we calculated the number of participants where both a diagnosis of mental illness or challenging behaviours or a neurodevelopmental disorder and a psychotropic medication prescription were recorded simultaneously. We then calculated this figure against the rate of the total number of participants who received specific psychotropic medications. On that basis, we calculated the proportion of participants for whom different psychotropic medications were used in association with various diagnoses. The same method had been used in previous epidemiological studies of the psychotropic prescription prevalence in adults with intellectual disabilities [8, 10].

### Statistical analysis

The data were processed using the Vertica 9 database and analysed using SPSS 29.0.1.0 [20] and STATA/IC 15.1 [21].

Absolute and relative frequencies were used as standard descriptive statistics for categorical data, including odds ratios (ORs) with 95% confidence intervals (CIs) and p-values. The incidence rate ratio (IRR) with 95% confidence interval (CI) and its statistical significance (p-values) were adopted to compare the incidence of new mental illness diagnosis, challenging behaviour, neurodevelopmental disorders, and psychotropic prescriptions between the years 2015 and 2022. Binary logistic regression analyses were used to determine factors influencing the occurrence of psychotropic medication, mental illness, and challenging behaviour among adults with intellectual disabilities. We estimated models using the stepwise forward conditional method. If adults with intellectual disabilities received a new prescription of psychotropic medication or a diagnosis of a mental illness or challenging behaviour after cohort entry (that is, during follow-up), we considered them no longer at risk for that psychotropic prescription or mental illness or challenging behaviour and removed them from the cohort.

We conducted multivariate regression analysis using the incidences of mental illnesses as dependent variables and age, gender, severity of intellectual disabilities, and incidence of challenging behaviour as the independent variables. We also conducted multivariate regression analysis using the incidences of new prescriptions of different psychotropic medications as dependent variables and gender, age, severity of intellectual disabilities, and incidences of challenging behaviour and different mental illnesses as the independent variables. We used Wald tests to assess the overall significance of categorical variables and categorical interaction terms. We considered a p-value of 0.05 to be statistically significant (two-tailed).

### Patient and public involvement

Children of the Full Moon, a non-governmental parent organisation for people with autism spectrum disorder and intellectual disabilities in the Czech Republic, has been involved at every stage of the study, from conceptualisation through design, data analysis, and paper writing. The organisation’s chairman (PT), also the parent of a child with autism, is a co-author.

## Results

Table 1 shows the demographic characteristics of all adults with a diagnosis of intellectual disabilities in 2022 (*n* = 62,636), new cases of adults with intellectual disabilities who showed challenging behaviour (*n* = 2147), and those receiving new prescriptions of any psychotropic medications in 2022 (*n* = 2398). Among those who had intellectual disabilities in 2022, and also among new cases of challenging behaviour in 2022 and who received any newly prescribed psychotropic medications in 2022, there were more male than female participants, more participants were older than 40 years than younger than 40, and with mild and moderate intellectual disabilities than severe and profound (see Table 1).

**Table 1:** Characteristics of the study population and psychotropic medicine prescription

All adults with intellectual disabilities in 2022 (*n* = 62,636) (F70-74)	
Gender	
Male	34 014 (54%)
Female	28 622 (46%)
Age group	
< 40 years	29 226 (47%)
40–64 years	24 453 (39%)
≥ 65 years	8 957 (14%)
The severity of intellectual disabilities	
Mild and moderate	49 032 (78%)
Severe and profound	7 998 (13%)
Other and unspecified	5 606 (9%)
**Incidence of those who displayed challenging behaviour in 2022 (F70x.1 and/or x.8) (n = 2147)**	
Gender	
Male	1 137 (53%)
Female	1 010 (47%)
Age group	
< 40 years	915 (42.6%)
40–64 years	843 (39.3%)
≥ 65 years	389 (18.1%)
The severity of intellectual disabilities	
Mild and moderate	1 791 (83.4%)
Severe and profound	307 (14.3%)
Other and unspecified	49 (2.3%)
**Incidence of any new psychotropic medication prescription in 2022 (n = 2, 398)**	
Gender	
Male	1 320 (55%)
Female	1 078 (45%)
Age group	
< 40 years	1 115 (46.5%)
40–64 years	861 (35.9%)
≥ 65	422 (17.6%)
The severity of intellectual disabilities	
Mild and moderate	1 851 (77.2%)
Severe and profound	271 (11.3%)
Other and unspecified	276 (11.5%)

**Fig. 1:**
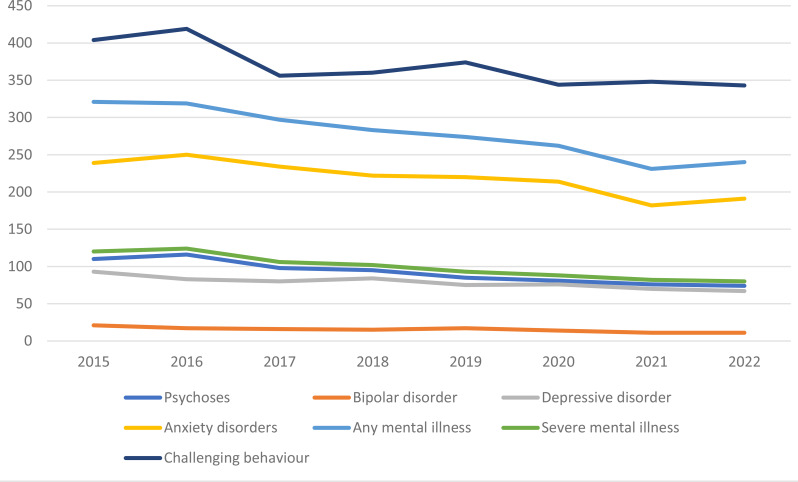
Incidence of mental illness and challenging behaviour per 10,000 person-years of adults with intellectual disabilities between 2015 and 2022

Table 2 shows the prevalence (2010 and 2022) and the incidence rates of new cases (2015 and 2022) of different mental illnesses, challenging behaviours and neurodevelopmental disorders like autism and ADHD. In 2010, 53,551 adults had a diagnosis of intellectual disabilities. This figure increased to 58,678 in 2015 and 62,636 in 2022. The incidence rate is presented per 10,000 person-years to compare data with the previous English study [8]. Figure 1 illustrates the trajectory of new cases recorded for various mental illnesses and challenging behaviours from 2015 to 2022, showing a decline in the incidence of new cases over the years, except for a slight increase in new cases of some mental illnesses and challenging behaviours in 2016, followed by a continuing decrease over the next few years.

**Table 2:** Prevalence (2010–2022) and incidence (2015–2022) of mental illness, challenging behaviour and neurodevelopmental disorders

Diagnosis	Prevalence (*N *= 53,551) 2010 *N* (%)	Prevalence (*N* = 62,636) 2022 *N* (%)	OR (95% CI), *p* values for prevalence between 2010 and 2022	Incidence per 10,000 person-years (*N* = 58,678) 2015	Incidence per 10,000 person-years (*N* = 62,636) 2022	IRR (95% CI) *p*-values for incidence between 2015 and 2022
Any mental illness	8419 (15.7%)	10,819 (17.3%)	1.12 (1.08–1.15), < 0.001	321	240	1.34 (1.25–1.43) *p* < 0.001
Severe mental illness	3938 (7.4%)	4835 (7.7%)	1.05 (1.01–1.10), < 0.02	120	80	1.50 (1.34–1.69) *p* < 0.001
Psychoses	3773 (7%)	4560 (7.3%)	1.04 (0.99–1.08), 0.123	110	74	1.05 (1.01–1.09) *p* < 0.02
Bipolar disorder	217 (0.4%)	327 (0.5%)	1.29 (1.09–1.53), < 0.01	22	11	1.92 (1.42–2.61) *p* < 0.001
Depressive disorder	1363 (3%)	1754 (3%)	1.10 (1.03–1.09), < 0.01	93	67	1.40 (1.23–1.59) *p* < 0.001
Anxiety disorders	3956 (7.4%)	5300 (9%)	3.54 (3.33–3.76), < 0.001	239	191	1.25 (1.16–1.35) *p* < 0.001
Autism	370 (0.7%)	1854 (3%)	4.38 (3.92–4.90), < 0.001	17	26	0.63 (0.49–0.82) *p* < 0.001
ADHD	244 (0.5%)	937 (2%)	3.32 (2.88–3.82), < 0.001	11	18	0.62 (0.45–0.85) *p* < 0.003
Challenging behaviour	15,512 (29%)	19,094 (30.5%)	1.08 (1.05–1.10), < 0.001	404	343	1.18 (1.11–1.25) *p* < 0.001

CI: confidence interval; IRR: incidence rate ratio; OR: Odds ratio

In 2010, the prevalence of any concomitant mental illness was 15·7%, and challenging behaviour 29%, increasing by 2022 to 17·3%, and 30·5%, respectively. The prevalence of individual mental illnesses remained similar or increased marginally between 2010 and 2022, except for anxiety disorder, which increased significantly over the same period (Odds Ratio (OR): 3.54; 95% Confidence Interval (CI): 3.33–3.76; *p* < 0.001) (see Table 2). Similarly, there was a significant increase in the prevalence of neurodevelopmental disorders like autism (OR: 4.38; 95% CI: 3.92–4.90; *p* < 0.001) and ADHD (OR: 3.32; 95% CI: 2.88–3.82; *p* < 0.001) between 2010 and 2022 (see Table 2). On the other hand, the incidence of newly diagnosed cases of most mental illnesses and challenging behaviour decreased between 2015 and 2022, except for autism and ADHD, which increased significantly during the same period.

Table 3 shows the prevalence (2010 and 2022) and incidence (2015 and 2022) data on the overall prescription of psychotropic medications and the rate of psychotropic medications prescribed among participants with challenging behaviour. We also presented in Table 3 prevalence data (2010 and 2022) on the proportion of those who received different psychotropic medications with different mental illness categories or challenging behaviours.

**Table 3:** Prevalence (2010–2022) and incidence (2015–2022) of psychotropic prescribing

The overall rate of psychotropic prescription							
Psychotropics	Prevalence (*N* = 53,551) 2010 *N* (%)	Prevalence (*N* = 62,636) 2022 *N* (%)	Odds ratio (95% confidence interval) *p*-values for prevalence rates between 2010 and 2022	Incidence per 10,000 person-years (*N* = 58,678) 2015	Incidence per 10,000 person-years (*N* = 62,636) 2022		IRR (95% CI) *p*-values for incidence rates between 2015 and 2022
Any psychotropic	29,616 (55%)	36,981 (59%)	1.16 (1.14–1.19) *p* < 0.001	457	383		1.19 (1.13–1.26) *p* < 0.001
Antipsychotics	18,808 (35%)	23,001 (37%)	1.07 (1.05–1.10) *p* < 0.001	317	257		1.23 (1.15–1.32) *p* < 0.001
Antidepressants	9,397 (17%)	15,847 (25%)	1.59 (1.55–1.64)* p* < 0.001	334	278		1.20 (1.12–1.28) *p* < 0.001
Anxiolytics	7,705 (14%)	7,953 (13%)	0.87 (0.84–0.90) *p* < 0.001	326	297		1.10 (1.03–1.17)* p* < 0.005
Hypnotics/sedatives	290 (0.5%)	635 (1%)	1.88 (1.64–2.16)* p *< 0.001	51	71		0.73 (0.62–0.84)* p* < 0.001
Mood stabilisers	12,158 (23%)	17,488 (28%)	1.32 (1.28–1.35) *p* < 0.001	379	368		1.03 (0.97–1.09) *p* = 0.35
**The rate of psychotropics prescribed for challenging behaviour**							
Psychotropics	Prevalence (*N* = 15,512) 2010 *N* (%)	Prevalence (*N*= 19,094) 2022 *N* (%)	Odds ratio (95% CI) *p*-values	Incidence per 10,000 person-years (*N* = 17,015) 2015	Incidence per 10,000 person-years (*N* = 19,094) 2022		IRR (95% CI) *p*-values for incidence rates between 2015 and 2022
Any psychotropic	12,689 (82%)	16,601 (87%)	1.48 (1.40–1.57) *p* < 0.001	507	403		1.26 (1.14–1.39) *p* < 0.001
Antipsychotics	9,590 (62%)	12,078 (63%)	1.26 (1.21–1.31) *p* < 0.001	512	427		1.20 (1.09–1.32) *p* < 0.001
Antidepressants	3,098 (20%)	6,378 (33%)	2.06 (1.96–2.16) *p* < 0.001	476	413		1.15 (1.04–1.27) *p* < 0.01
Anxiolytics	2,693 (17%)	2,658 (14%)	0.77 (0.73–0.82) *p* < 0.001	353	338		1.04 (0.93–1.17) *p* = 0.45
Hypnotics/sedatives	68 (0.4%)	213 (1%)	2.56 (1.95–3.37) *p* < 0.001	45	74		0.61 (0.45–0.81) *p* < 0.001
Mood stabilisers	4,634 (30%)	6,987 (37%)	1.35 (1.29–1.42)* p* < 0.001	370	414		0.89 (0.80–0.99) *p* < 0.05
**The rate of mental illnesses and challenging behaviour among those who received different psychotropic medications**							
**Mental illness**			**Challenging behaviour**				
Psychotropics	2010	2022	Psychotropics	2010		2022	
Antipsychotics in severe mental illness	3475 (18%)	4440 (19%)	Antipsychotics	9590 (51%)		12078 (52%)	
Antidepressants in depressive disorder	996 (11%)	1300 (8%)	Antidepressants	3098 (33%)		6378 (40%)	
Anxiolytics in anxiety disorders	1232 (16%)	1481 (19%)	Anxiolytics	2693 (35%)		2658 (33%)	
Hypnotics/sedatives in sleep disorders	0	2	Hypnotics/sedatives	68 (23%)		212 (33%)	
Mood stabilisers in bipolar disorder	137 (1%)	204 (1%)	Mood stabilisers	4634 (38%)		6987 (40%)	

CI: confidence interval; IRR: incidence rate ratio

The prevalence of psychotropic medication prescribing among adults with intellectual disabilities increased from 55% in 2010 to 59% in 2022. Similarly, antipsychotic prescribing increased from 35% in 2010 to 37% in 2022, and antidepressants from 17% in 2010 to 25% in 2022. The prevalence of mood stabiliser prescriptions also increased from 23% in 2010 to 28% in 2022, and hypnotics/sedatives from 0.5% in 2010 to 1% in 2022. The rise in the prevalence of hypnotics/sedatives (OR: 1.88; 95% CI: 1.64–2.16; *p* < 0.001) and antidepressants (OR: 1.59; 95% CI: 1.55–1.64; *p* < 0.001) was likely to be significant. In contrast, the prevalence of anxiolytic prescribing fell from 14% in 2010 to 13% in 2022 (OR: 0.87; 95% CI: 0.84–0.90; *p* < 0.001).

Among those who showed challenging behaviour, a high proportion received psychotropic medications (82%) in 2010, increasing in 2022 to 87%. A high proportion also received antipsychotic medications (62%) in 2010, which increased in 2022 to 63%. However, the prevalence of antidepressant prescriptions among those who displayed challenging behaviour increased significantly from 20% in 2010 to 33% in 2022 (OR: 2.06; 95% CI: 1.96–2.16; *p* < 0.001), and so did the hypnotics/sedatives from 0.4% in 2010 to 1% in 2022 (OR: 2.56; 95% CI: 1.95–3.37; *p* < 0.001). In contrast, the prevalence of anxiolytic prescriptions among those displaying challenging behaviour decreased from 17% in 2010 to 14% in 2022 (OR: 0.77; 95% CI: 0.73–0.82; *p* < 0.001).

**Fig. 2:**
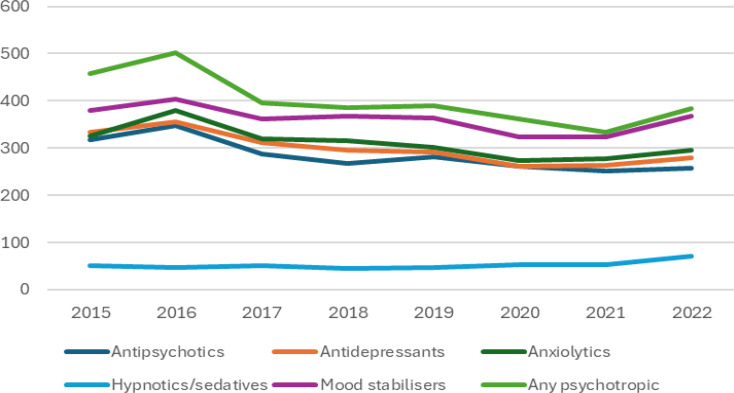
Incidence of new psychotropic prescriptions between 2015 and 2022 per 10,000 person-years of adults with intellectual disabilities

The incidence rates of new cases of psychotropic prescriptions are presented in Table 3 and Fig. 2. The incidence rates decreased from 2015 to 2022 for most psychotropic medication prescriptions, except for hypnotics/sedatives and mood stabilisers, which increased between 2015 and 2022. The increase in the hypnotics/sedatives was statistically significant (incident rate ratio (IRR): 0.61; 95% CI: 0.45–0.81; *p* < 0.001). The trajectory showed a rise in incidence in most psychotropics in 2016, followed by a subsequent decrease in 2017. However, there was another rise in the incidence rates of most psychotropic medications in 2022 (the last year of data collection) (see Fig. 2).

**Fig. 3:**
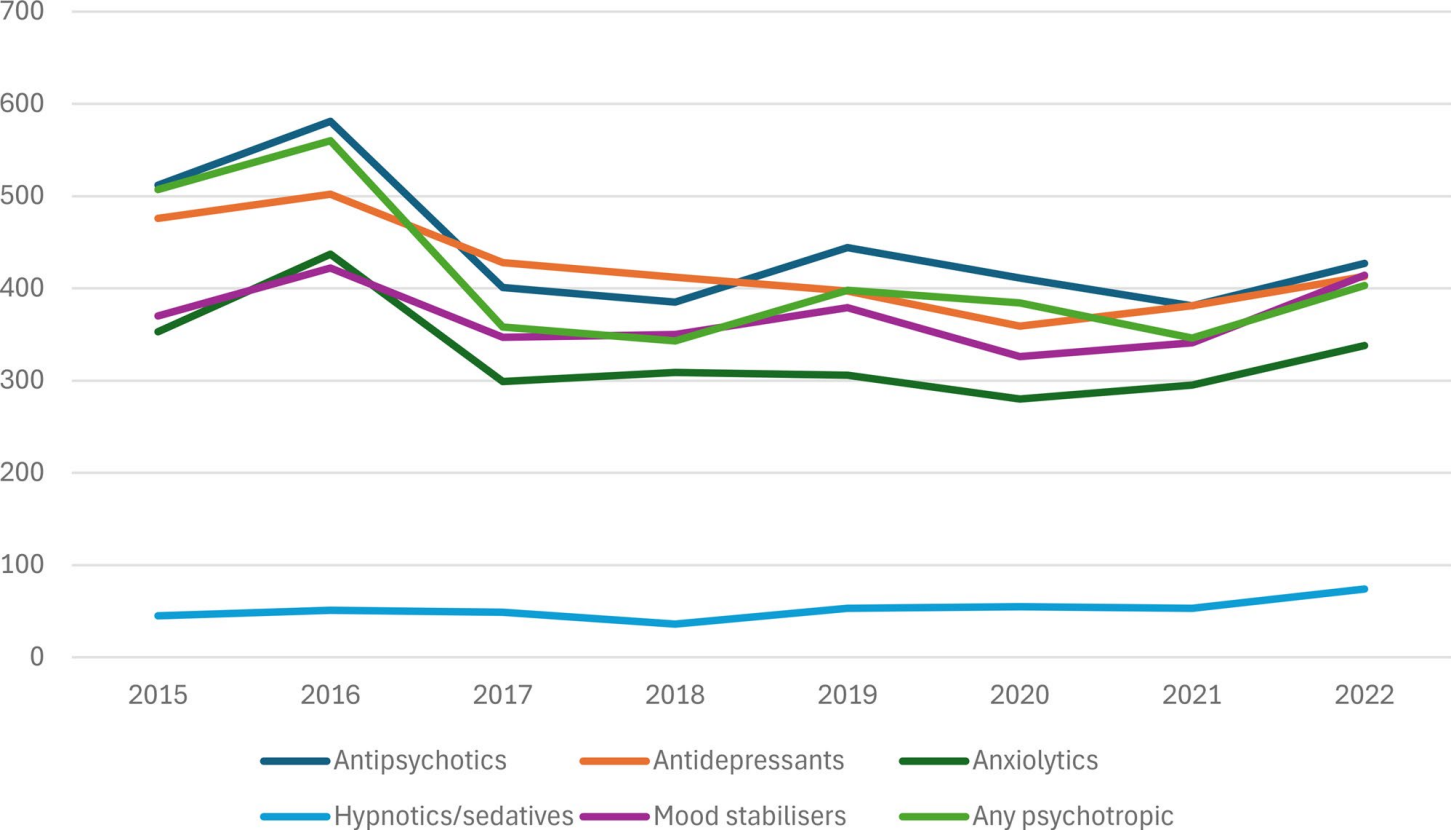
Incidence of new prescriptions of psychotropic medications for challenging behaviour in adults with intellectual disabilities between 2015 and 2022 per 10,000 person-years

Whereas the incidence rate of new prescriptions of antipsychotics, antidepressants and anxiolytics decreased between 2015 and 2022 for those who displayed challenging behaviour, the rate of new prescriptions of hypnotics/sedatives and mood stabilisers increased over the same period (see Table 3). Figure 3 shows that the incidence rates of new prescriptions for psychotropic medications in participants with challenging behaviour increased during 2016 and again in 2019 and 2022, with corresponding decreases in the following years (except 2022, which was the last year of data collection).

Among those who received antipsychotics, only 18% in 2010 and 19% in 2022 had a concomitant diagnosis of severe mental illness (schizophrenia and other psychoses and/or bipolar disorder) for which they are indicated (see Table 3). This means that in over 80% of cases, antipsychotics were prescribed when there was no severe mental illness present. On the contrary, among those who received antipsychotics, 51% in 2010 and 52% in 2022 had a diagnosis of challenging behaviour. There was an association between antidepressant prescription and depressive disorders in only 11% in 2010 and 8% of cases in 2022. On the other hand, there was an association between antidepressant prescribing and challenging behaviour in 33% of cases in 2010 and 40% in 2022. Similarly, there was an association between anxiolytic prescription and anxiety disorders only in 16% in 2010 and 19% of cases in 2022, but challenging behaviour in 35% of cases in 2010 and 33% in 2022. Similarly, there was an association between mood stabiliser prescription and challenging behaviour diagnosis in 38% of cases in 2010 and 40% in 2022. In contrast, mood stabiliser prescription was associated with only 1% of cases of bipolar disorder in 2010 and 2022 (see Table 3).

Table 4 shows the results of multivariate regression analysis. The incidence of challenging behaviour correlated significantly with the incidence of psychoses, bipolar disorder, and anxiety disorders.

The female gender was significantly associated with the incidences of bipolar disorder (*p* < 0.005), depressive disorder (*p* < 0.001), and anxiety disorder (*p* < 0.001). Older age was significantly related to the incidence of psychoses (*p* < 0.05), depressive disorder (*p* < 0.001), and anxiety disorder (*p* < 0.001). Severe and profound intellectual disabilities were significantly associated with the incidence of psychoses (*p* < 0.001) and anxiety disorders (*p* < 0.005).

**Table 4:** Factors influencing the occurrence of incidence of different mental illnesses and challenging behaviour among adults with intellectual disabilities (F70-F79) (multivariate analysis)

		Psychoses (F20-29)		Bipolar disorder (F30-31)		Depressive disorder (F32-39)		Anxiety disorder (F40-48)	
		OR (95% CI)	*p*	OR (95% CI)	*p*	OR (95% CI)	*p*	OR (95% CI)	*p*
Gender	Women	N/A	N/A	1.259 (1.090; 1.454)	0.002	1.507 (1.413; 1.608)	< 0.001	1.337 (1.286; 1.390)	< 0.001
Age	< 40	reference		reference		reference		reference	
	40–64	1.121 (1.015; 1.239)	0.025	N/A	N/A	1.240 (1.114; 1.380)	< 0.001	1.689 (1.581; 1.804)	< 0.001
	≥ 65	1.154 (1.044; 1.276)	0.005	N/A	N/A	1.371 (1.233; 1.523)	< 0.001	1.259 (1.177; 1.347)	< 0.001
The severity of ID	Mild and moderate	reference		reference		reference		reference	
	Severe and profound	2.684 (2.243; 3.212)	< 0.001	1.231 (0.913; 1.658)	0.173	1.097 (0.980; 1.229)	0.109	0.904 (0.845; 0.968)	0.004
	Other and unspecified	1.172 (0.954; 1.442)	0.131	0.889 (0.620; 1.276)	0.524	0.565 (0.482; 0.661)	< 0.001	0.391 (0.354; 0.432)	< 0.001
Challenging behaviour		2.529 (2.382; 2.686)	< 0.001	2.085 (1.796; 2.420)	< 0.001	N/A	N/A	1.129 (1.082; 1.179)	< 0.001

CI = Confidence Interval, N/A = Not applicable (no significant association was found), OR = Odds ratio, and p = *p*-values

Table 5 presents the findings from a multivariate regression analysis, using the incidence of new psychotropic prescribing as the dependent variable. The rate of challenging behaviour was significantly associated with the rate of prescription of any psychotropic medication (*p* < 0.001), antipsychotics (*p* < 0.001), antidepressants (*p* < 0.001), and antianxiety medications (*p* < 0.001). The female gender was statistically significantly associated with the prescription of antidepressants and antianxiety medications, but not antipsychotics and mood stabilisers. Older age (> 65 years) was statistically significantly related to the prescription of all psychotropic medications. The more severe intellectual disabilities (severe and profound intellectual disabilities) were statistically significantly associated with the prescription of all psychotropics apart from antipsychotic medication. Anxiety disorder was statistically significantly associated with all psychotropic medication prescriptions.

**Table 5:** Factors influencing the incidence of psychotropic medication prescribing among adults with intellectual disabilities (F70-F79) (multivariate analysis)

		Antipsychotics		Antidepressants		Anxiolytics		Hypnotics and sedatives		Any psychotropic	
		OR (95% CI)	*p*	OR (95% CI)	*p*	OR (95% CI)	*p*	OR (95% CI)	*p*	OR (95% CI)	*p*
Gender	Women	N/A	N/A	1.143 (1.105; 1.182)	< 0.001	1.163 (1.126; 1.202)	< 0.001	N/A	N/A	N/A	N/A
Age	< 40	reference		reference		reference		reference		reference	
	40–64	0.729 (0.694; 0.767)	< 0.001	0.920 (0.873; 0.970)	0.002	1.092 (1.036; 1.152)	0.001	0.545 (0.485; 0.612)	< 0.001	1.035 (0.989; 1.083)	0.138
	≥ 65	0.688 (0.655; 0.724)	< 0.001	0.825 (0.783; 0.870)	< 0.001	1.098 (1.042; 1.158)	< 0.001	0.788 (0.706; 0.880)	< 0.001	0.937 (0.895; 0.980)	0.005
The severity of ID	Mild and moderate	reference		reference		reference		reference		reference	
	Severe and profound	N/A	N/A	0.923 (0.868; 0.982)	0.011	0.803 (0.760; 0.849)	< 0.001	0.621 (0.552; 0.699)	< 0.001	0.853 (0.812; 0.895)	< 0.001
	Other and unspecified	N/A	N/A	0.747 (0.690; 0.807)	< 0.001	0.712 (0.663; 0.766)	< 0.001	0.593 (0.507; 0.693)	< 0.001	0.783 (0.735; 0.834)	< 0.001
Challenging behaviour		2.198 (2.124; 2.276)	< 0.001	1.892 (1.827; 1.960)	< 0.001	1.101 (1.062; 1.142)	< 0.001	N/A	N/A	1.167 (1.130; 1.205)	< 0.001
Psychoses		1.357 (1.285; 1.434)	< 0.001	1.184 (1.119; 1.254)	< 0.001	1.107 (1.044; 1.173)	0.001	N/A	N/A	0.728 (0.685; 0.774)	< 0.001
Bipolar disorder		1.287 (1.087; 1.524)	0.003	N/A	N/A	N/A	N/A	N/A	N/A	0.739 (0.600; 0.911)	0.005
Depressive disorder		1.694 (1.572; 1.825)	< 0.001	1.854 (1.729; 1.987)	< 0.001	1.522 (1.412; 1.641)	< 0.001	1.356 (1.121; 1.640)	0.002	N/A	N/A
Anxiety disorder		2.602 (2.487; 2.722)	< 0.001	3.259 (3.126; 3.398)	< 0.001	2.216 (2.119; 2.316)	< 0.001	2.035 (1.823; 2.272)	< 0.001	1.606 (1.538; 1.678)	< 0.001

CI = Confidence Interval, N/A = Not applicable (no significant association was found), OR = Odds ratio, and p = *p*-values

## Discussion

### Mental illness, neurodevelopmental disorders and challenging behaviour

The overall prevalence of mental illness, most individual mental illness categories, and challenging behaviour, which was high at study entry in 2010, remained the same or increased marginally over 12 years between 2010 and 2022, except anxiety disorder and the neurodevelopmental disorders like autism and ADHD, all of which increased significantly during the same period. The reported prevalence of mental illnesses, neurodevelopmental disorders, and challenging behaviour among adults with intellectual disabilities varied widely in previous studies for obvious methodological reasons [5]. Ours is the first large national health register-based study that used ICD diagnosis for adults with intellectual disabilities, a method that had not been employed previously in a large national health database. However, the overall prevalence of mental illness and challenging behaviour found in the current study is similar to what was reported in some previous studies [5]. For example, a whole population-based cohort study of adults with intellectual disabilities in Scotland found the overall prevalence [22] of mental illness and severe mental illness (psychoses and bipolar disorder) among 16.3% and 6.2% of the cohort (*n* = 1190), respectively, at study entry in 2002–2004 compared with 15.7% and 7.4% respectively in the current study at study entry in 2010 (*n* = 53,551). Similarly, in the Scottish study, depressive disorder was diagnosed among 4.3%, anxiety disorder in 2.7%, autism in 7.6%, and ADHD in 1.3% of the cohort, compared with 3%, 9%, 3%, and 2%, respectively, in the current study in 2022.

The reported prevalence of challenging behaviour varied widely in previous studies because of the different methodologies used. A significant problem has been defining and diagnosing challenging behaviour in this population. The current study employed an ICD-10 diagnosis, which has not been used in previous studies, to diagnose challenging behaviour, despite the fact that ICD-10 provides an explicit qualifier with an extended ‘x’ code for this diagnosis. Despite these methodological differences, the prevalence of challenging behaviour reported in the current study is similar to some earlier studies [5, 6]. For example, in the Scottish population-based study [22], 20.5% of the cohort at study entry displayed challenging behaviour, compared with 29% in the current study at study entry. However, the incidence rate of new challenging behaviour cases fell by 0·6% from 404 per 10,000 person-years in 2015 to 343 in 2022. An increased prevalence rate of challenging behaviour over the years, despite the decrease in the incidence rate, may be due to the chronic nature of challenging behaviour. Although the incidence rates of mental illnesses and challenging behaviour decreased between 2015 and 2022, there was a significant increase in the newly diagnosed cases of neurodevelopmental disorders, such as autism and ADHD, during the same period. This is in keeping with the current trend of increased incidence rates of autism and ADHD in general, but also because the clinicians are assessing for and recognising other concomitant neurodevelopmental disorders more often among adults with intellectual disabilities [23, 24].

### Psychotropic medication prescribing

#### The prevalence of psychotropic medication prescribing among adults with intellectual disabilities between 2010 and 2022

The reported prevalence rates of psychotropic medication prescribing for adults with intellectual disabilities in previous studies varied from as low as 10% to as high as 67%, depending on the method of data collection [10, 11]. In a recent meta-analysis of 24 studies, Song and colleagues (2023) reported a pooled prevalence of psychotropic prescribing in adults with intellectual disabilities. Pooled prevalence for any psychotropic prescription was 41% (95% CI: 35–46%), antipsychotics 31% (95% CI: 27–35%), antidepressants 14% (95% CI: 9–19%), anxiolytics 9% (95% CI: 4–15%), and hypnotics/sedatives 5% (95% CI: 2–8%) [10]. This shows that apart from the prevalence of any psychotropic medications and hypnotics/sedatives prescriptions among adults with intellectual disabilities in the current study, the prevalence rates of other psychotropic medication prescriptions at the study entry were within the 95% confidence intervals reported in the recent meta-analysis [10]. In the English general practice register-based study of 33,016 adults with intellectual disabilities [8], the rate of any psychotropic prescription was 49% at study entry in 1999 and 63% at the study end in 2013. This compares with the 55% prevalence of any psychotropic prescription in 2010 and 59% in 2022 in the current study. The rate of increase in psychotropic medication prevalence in our study over 12 years was lower than that in the English study over 15 years. The most commonly prescribed psychotropic medication was antipsychotics in 2022 (37%), followed by mood stabilisers, primarily antiepileptics (28%), antidepressants (25%), anxiolytics (13%) and hypnotics/sedatives (1%). This compares with a recent study among adults with intellectual disabilities in community homes and supported living accommodations in England and Wales in the UK, which reported that of 190 prescriptions, 47% were antipsychotics, followed by 23% antidepressants, 13% antiepileptics, and 7% anxiolytics in the form of benzodiazepine [25].

The prevalence of antipsychotic medication prescription among adults with intellectual disabilities in the current study was much higher than in the general population, which is less than 3% [26]. Whereas 35–37% of the adults with intellectual disabilities were prescribed antipsychotics in the current study, only 7.4–7.7% had a diagnosis of psychosis and/or bipolar disorder (severe mental illness) for which antipsychotics are licensed. This shows that in the vast majority of cases, antipsychotics were not prescribed for severe mental illness. The prevalence of antidepressant prescribing in the current study was much higher than in the general population, which is 10.3% [2]. Whereas 17–25% of the adults with intellectual disabilities were prescribed antidepressants, only 3% had a diagnosis of depressive disorder in the current study, for which antidepressants are licensed. This shows that in the vast majority of cases, antidepressants were not prescribed among participants with a diagnosis of depressive disorder. However, due to a lack of space, we did not present data on the association between antidepressant prescribing and their other indications apart from depression, such as anxiety disorders.

#### The incidence of new psychotropic prescriptions among adults with intellectual disabilities between 2015 and 2022

The incidence of new prescriptions of any psychotropic medications was 503 in 1999 and 533 per 10,000 person-years in 2013 in the English study [8], compared with the incidence rate of new psychotropic medications prescriptions of 457 in 2015 and 383 per 10,000 person-years in 2022 in the current study, which was lower than the rates in the English study at both data entry points. Whereas the English study [8] showed an increase in the incidence rate of new prescriptions for most psychotropic medications over 15 years, the current study showed a decrease in the incidence rate of most psychotropic medication prescriptions over seven years, except for a significant increase in hypnotic/sedative prescriptions. Despite this decrease in the incidence rate in the current study, the prevalence of antidepressants increased between 2010 and 2022. This is likely due to the long-term use of these medications. The reasons for this require urgent scrutiny to prevent inappropriate long-term prescribing of psychotropic medications in adults with intellectual disabilities.

However, Fig. 2 shows a rise in incidences in almost all psychotropic medications around 2016, followed by a subsequent fall in 2017, and another rise in 2022, which is the last year of data collection in the current study. This increase is difficult to explain and may not directly relate to the COVID-19 effect [27]. A similar trend was reported in the English study [8], in that after a reduction in 2010-11 in the incidence of new prescriptions of anxiolytics and antidepressants, there was a subsequent increase in 2012-13.

#### The prevalence of psychotropic medication prescribing between 2010 and 2022 among adults with intellectual disabilities who displayed challenging behaviour

The prevalence of antipsychotic prescriptions among those who displayed challenging behaviour in the current study was higher than that reported in the English study [8]. For example, 47% of those with challenging behaviour received antipsychotics in the English study [8] compared with 62% in 2010 and 63% in 2022 in the current study. One possible explanation is that there is a relative lack of availability of non-pharmacological interventions for supporting adults who display challenging behaviour in the Czech Republic compared to the UK.

In previous studies, among those who received psychotropic medications, most received them outside their licensed indications [7, 8, 11, 25, 28]. For example, in the current study, antipsychotics were prescribed for severe mental illness only in 18% of cases in 2010 and 19% in 2022, compared with 24% in the Norwegian [28], 22% in the Dutch [7], and 29% in the English study [8]. A similar trend has recently been reported among adults with intellectual disabilities in community settings in England and Wales, where antipsychotics were prescribed for severe mental illness (psychoses and bipolar disorder) only in 19% of cases [25]. This means that in over 80% of cases, when antipsychotics were prescribed off-label for adults with intellectual disabilities, there was no record of any severe mental illness (psychoses and/or bipolar disorder) for which antipsychotics are indicated. However, among those who received antipsychotic medications, the proportion for whom they were used among those who displayed challenging behaviour in the current study was lower (51–52%) than the Dutch study (58%) [7] and the English study (61%) [8]. Like the previous studies [7, 8, 25], the current study clearly showed that antipsychotic medications were prescribed primarily off-license among adults with intellectual disabilities who displayed challenging behaviour rather than mental illnesses, such as those with psychoses or bipolar disorder, for which they are licensed.

A similar trend was observed for other psychotropic medications in the current study. For example, antidepressants were prescribed in the presence of a diagnosis of depressive disorder only in 8–11% of cases, but were prescribed more often among those displaying challenging behaviours (33–40% of cases). Similarly, mood stabilisers were prescribed in the presence of bipolar disorder only in 1% of cases, whereas they were prescribed among those displaying challenging behaviour in 38–40% of cases (see Table 3). This compares with a recent community-based study of adults with intellectual disabilities, which showed antiepileptic medications were used for epilepsy less often (in 32% of cases) than challenging behaviour (in 40% of cases) [25]. This shows that psychotropic medications are prescribed off-licence in a high proportion of cases, particularly among those displaying challenging behaviour in adults with intellectual disabilities, against the NICE and the WPA guidelines recommendations [15, 11, 16]. However, the off-licence use of a licensed medication is not necessarily inappropriate if appropriate safeguards are implemented [29]. Due to a lack of space, we did not present the association between benzodiazepines for other indications beyond anxiety disorder, such as sleep problems. The significant rise in the prevalence rate of antidepressant prescribing is in keeping with what has been recently reported from the UK [30].

Despite the significant increase in the prevalence of anxiety disorders, in the current study, between 2010 and 2022, the decrease in the prevalence of anxiolytic prescribing during the same period may be difficult to explain. However, one possible explanation is that clinicians are using antidepressants like SSRIs more often to treat anxiety disorders than anxiolytics. This is indirectly supported by the significant increase in antidepressant prescribing over the same period in the current study.

#### The incidence of new psychotropic medication prescriptions among adults with intellectual disabilities between 2015 and 2022 who displayed challenging behaviours

However, the incidence of new prescriptions among those who displayed challenging behaviour showed that, despite a decrease in most psychotropic prescriptions, there was a significant increase in hypnotics/sedatives and a marginally significant increase in mood stabiliser prescriptions between 2015 and 2022. This shows that in the current study, the welcome decrease in new prescriptions for antipsychotics, antidepressants, and anxiolytics prescribed among participants who displayed challenging behaviour may have been achieved at the expense of a not-so-welcome rise in other psychotropic prescriptions, particularly hypnotics/sedatives and possibly mood stabilisers. An English paper from the UK reported a recent trend in increased antidepressant prescribing to compensate for the reduction in antipsychotic prescribing [30].

### Relationship among mental illnesses, challenging behaviour and psychotropic prescribing

The findings of a significant association among challenging behaviour and psychoses, bipolar disorder, and anxiety disorder (see Table 4) are similar to what has been reported previously [5, 6]. The incidence of psychoses and anxiety disorders showed a significant association with more severe intellectual disabilities. However, diagnosis of mental illnesses, particularly schizophrenia, is unreliable in adults with severe and profound intellectual disabilities [see 5 for a review]. Similarly, challenging behaviour may have been diagnosed as anxiety in some adults with intellectual disabilities [5, 6, 25]. This is reflected in the significant rise in anxiety disorder diagnoses between 2010 and 2022 in the current study.

### The relationship between psychotropic medication prescribing and other variables

Table 5 showed that the incidences of psychotropic medication prescriptions were significantly higher in the presence of mental illness, challenging behaviour, older age and more severe intellectual disabilities. A higher incidence of psychotropic prescribing in those with a mental illness and challenging behaviour had been reported before [7, 8, 11]. The significant association in the incidence of psychotropic medication prescribing and adults with severe intellectual disabilities and older age could have been a confounding effect of a similar association of age and severity of intellectual disabilities with mental illnesses and challenging behaviour per se. The fact that the female gender in the current study was statistically significantly associated with the prescription of antidepressants and anxiolytics but not antipsychotics and mood stabilisers is difficult to explain. It is possible that clinicians consider anxiety and depression as underlying causes of challenging behaviour, more so among women with intellectual disabilities than men. A statistically significant association between older adults with intellectual disabilities and psychotropic medication prescribing is in keeping with previous studies among older adults with intellectual disabilities [31].

### Strengths and limitations

This is the first-ever population-based cohort study using a large national health database and ICD-10 of incidence and prevalence of mental illness, challenging behaviour and psychotropic medication prescribing and their relationship in adults with intellectual disabilities. No other large national health records-based study has used ICD criteria to diagnose mental illness and challenging behaviour before. Only one similar study in England used Reed codes from a general practice database to diagnose mental illness and challenging behaviour, which did not correspond with ICD-10 codes. They also used the British National Formulary rather than the WHO ATC for psychotropic medication classification used in this study. Therefore, it is difficult to generalise their findings outside England. No other large-scale study exists outside England, particularly in Eastern Europe. This is also the first study to assess the impact of WPA international, NICE guidelines, and the STOMP initiative in England, UK, on psychotropic medication prescribing for adults with intellectual disabilities.

Capturing data from the whole adult population with intellectual disabilities will never be possible, as many adults with mild intellectual disabilities are not known to the services or do not receive a diagnosis of intellectual disabilities. In the current study, we aimed to be as inclusive as possible by capturing both primary and secondary diagnoses from psychiatric, medical, surgical, and other inpatient and outpatient settings, as well as laboratory and allied professional data. The total Czech adult population (18 years and older) was 8,688,840 in 2022 (data gathered from the Institute of Health Information and Statistics of the Czech Republic, provided by authors JJ and HM), and we captured data on 62,636 adults with intellectual disabilities. This gives a prevalence of 0·7% of intellectual disabilities in the Czech adult population. The prevalence of adults with intellectual disabilities is reported to be between 0·5% and 0·7% of the whole adult general population [1–3, 32]. The demographics of the intellectual disabilities population in 2022 in the current study showed a higher proportion (54%) of males than females, a younger age, and mild to moderate rather than severe/profound intellectual disabilities, all of which are in keeping with what would be expected from a population-based sample of adults with intellectual disabilities [1, 3]. Considering all these factors, we believe our sample is as representative of the total adult Czech population with intellectual disabilities as possible. As the source data did not provide an indication for prescribing each psychotropic medication, we had to calculate an association between psychotropic prescribing and mental illness, and challenging behaviour. Due to the large sample size, even a small change in the prevalence rates between 2010 and 2022 appeared statistically significant according to the p-values. However, this does not mean that these values are clinically relevant. This, however, shows a general trend in the prescribing practice over ten years. To mitigate against this, we have presented additional statistics, including ORs with 95% CIs and IRRs with 95% CIs.

## Conclusion

The rate of challenging behaviour significantly correlated with mental illnesses. The rate of psychotropic medication prescribing significantly correlated with the rate of mental illness and challenging behaviour. There is a high prevalence of psychotropic medication use among adults with intellectual disabilities in the Czech Republic. Among those who displayed challenging behaviour, the prevalence of psychotropic medication was high. The rate increased for most psychotropics between 2010 and 2022, with a significant increase in antidepressant and hypnotics/sedatives prescriptions, except for anxiolytics, the rate of which fell during the same period. On the contrary, the incidence rates of new prescriptions of most psychotropic medications fell apart from hypnotics/sedatives, which increased significantly between 2015 and 2022 and mood stabilisers that increased with a marginal significance. Most psychotropic medications were prescribed only in a minority of cases in the presence of mental illnesses for which they are licensed. In a high proportion of cases, psychotropic medications were prescribed off-label among those who displayed challenging behaviour. The reasons for the high rates of psychotropic medication prescriptions among those who displayed challenging behaviour and an increase in most of them over 12 years, which goes against WPA and NICE guidelines, require stringent scrutiny to prevent future inappropriate overmedication of adults with intellectual disabilities, which is a significant public health concern. It is, however, worth emphasising that psychotropic medications play an essential role in the management of mental illness and challenging behaviour in adults with intellectual disabilities when used rationally, particularly if all non-pharmacological approaches prove ineffective and the patient poses a serious risk to themselves and others [15]. Therefore, the pros and cons of prescribing psychotropic medications in adults with intellectual disabilities have to be carefully assessed in each case. Population-based data can only show specific trends in national clinical practice.

## Data Availability

The datasets used and/or analysed during the current study are available from the corresponding author on reasonable request.

## Abbreviations

ADHD: Attention Deficit Hyperactivity Disorder
ATC: Anatomical Therapeutic Chemical
CI: Confidence Interval
ICD-10: International Classification of Diseases-10^th^ revision
ICD-11: International Classification of Diseases-11^th^ revision
IRR: The Incidence Rate Ratio
NHIS: National Health Information System
NHS: National Health Service
NICE: National Institute for Health and Care Excellence
NRRHS: National Registry of Reimbursed Health Services
PBS: Positive Behaviour Support
RCT: Randomised Controlled Trial
SPSS: Statistical Package for the Social Sciences
SSRI: Selective serotonin reuptake inhibitors
STOMP: STopping Over-Medication of People with ID, autism or both
UK: The United Kingdom
WHO: World Health Organization
WPA: The World Psychiatric Association
